# If the Coronavirus Doesn’t Scare You, the Banners Will—A Case Study of Early COVID-19 Banners

**DOI:** 10.3390/ijerph17249595

**Published:** 2020-12-21

**Authors:** Hongjie Dong, Minli Zhou, Dewei Che, Adams Bodomo

**Affiliations:** 1 School of Liberal Arts, Xi’an University, Xi’an 710065, China; 2 Department of Liberal Arts, Guangdong University of Education, Guangzhou 510303, China; minlizhou@163.com; 3 Faculty of Philological and Cultural Studies, University of Vienna, Vienna 1090, Austria; dewei.che@univie.ac.at (D.C.); adams.bodomo@univie.ac.at (A.B.)

**Keywords:** Chinese banners, mass communication, circulation mode, discourse style, adaptive adjustment

## Abstract

As a crucial element of China’s political and cultural life, “banners”, or biāoyǔ, have been around for decades, in support of national-level policies such as family planning and the governing mottos of Presidents. The banners that have emerged during the Covid-19 pandemic which was also the subject of a national-level driven policy, have been involved in a nation-wide public debate over the language styles of banners used to urge people to stay indoors. Based on the analysis of the early COVID-19 banners and the related online comments, this article analyzes the language style patterns of the banners and the mode of banner circulation. The study found that the manner in which the banners are circulated goes beyond a unidirectional path of on-site instant communication. This process is facilitated by social networks and mass media, which, during circulation, twice created a banner upgrade. The upgrades created decontextualization and function extension of the banners, whereas audience feedback triggered an adaptive adjustment of the language style of the banners. This article suggests that the study of the use and spread of banners, especially the early COVID-19 banners, sheds light on the study of mass communication and discourse style, and also how measures to contain pandemics such as COVID-19 can be communicated.

## 1. Introduction

An important aspect of the COVID-19 pandemic that merits discussion and documentation for posterity as to how the world contained the pandemic is the use of banners to enforce social distancing. As a crucial element of China’s political and cultural life, “banners”, or biāoyǔ in Chinese, have been around for decades, in support of national-level policies such as family planning, which started in 1962 and was updated in various phases until the present, and the governing ‘mottos’ of presidents. Being an important part of China’s public information system, together with government files, announcements, official notices etc., banners have long played an important role in public management as a propaganda tool [1]. During the fight against COVID-19, a huge number of banners emerged and spread rapidly through social media such as Weibo and WeChat. The banners that emerged during this epidemic were also triggered by a national-level driven policy, but, interestingly, this time a nation-wide public debate emerged over the language style used, particularly in the context of urging people to practice social distancing.

The lockdown of Wuhan city and the subsequent lockdown of the whole country demonstrated that China was facing a health crisis, now known worldwide as COVID-19. During the fight against this epidemic (which later on got upgraded into a pandemic by the World Health Organization), China’s public propaganda system focused on measures to control and diminish the impact of the coronavirus and as part of that effort a huge number of banners were designed and put into use [1]. Chinese state media like the People’s Daily, Guangming Daily, and China Central Television (CCTV) also reported on the banners, which accelerated their spread and prominence in both official and non-official settings. As it was put on the internet, “nothing is certain but death and banners”, which illustrates the importance of banners as a vast all-weather propaganda tool in Chinese society, used by the communist regime to disseminate ideas to the people. Through these efforts, a social language landscape was created, which can immediately be recognized as uniquely Chinese. Back in 1934, the “Red Star”, an important newspaper of the Red Army General Political Department, called on all soldiers to write banners to spread revolutionary ideology to the ordinary people. As demonstrated, banners have a long history in modern Chinese society and still serve as vibrant political symbols.

In order to understand better the nature and content of government-public communication during this unprecedented public health crisis, we conducted a survey of the language used in various types of banners aiming at documenting the development of these banners and analyzing the banner language in political, social, and cultural discourses that took place during the COVID-19 pandemic. The first question we addressed was the nature of the various slogans used. What kind of phraseology did authorities rely on most to influence the behavior of the public and which type engendered most comments on the social media? Another related question was the role of social media in the spread of the COVID-19 banners. How did online community members (netizens) react to the public banners and what was the result of their participation as regards the spread and content of the original messages? What is the extent to which authorities reacted to these online discussions and would they take notice of these public comments and therefore, alter some of their phraseologies or framings when met with general opposition or indignation as a result of the content of the banners?

In the paper, we will first give an overview of the banner tradition in China and discuss the main relevant studies and findings in that context. After that review, we outline the data collection method and also ensure that special terms are explained. Thereafter, we present the survey results, which are classified as official, gentle, humorous, and confrontational. In that section (i.e., the Results section), we also present data that clarify the effect of netizen’s online participation on the spread of COVID-19 banners. In the Discussion section, we look back at our research questions and present a model for the circulation of banner messages, which relates real-life banners with the online world of banner distribution and combines the level of banner creation by online readers, banner recreation in interaction with the general audience and a feedback loop to the original banner-making authorities.

**Figure 1:**
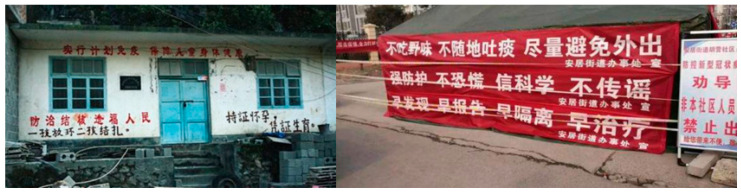
Banners on the wall of a house (**left**); Anti-Covid-19 banners (**right**).

Since Chinese banners are not a familiar phenomenon in western societies, it seems appropriate to introduce the Chinese banners in more detail here first. To begin with, ‘banners’ can appear at many different surfaces; they can, for instance, appear on well-situated buildings and as Figure 1 shows, COVID-19 banners, as a rule, have white characters on a red background. It further is important to note that in China, in the political domain, only governmental agencies have the right to create and post banners. Outside the political domain, in areas such as education, tourism, and commerce, but also during social protests and judicial disagreement the use of banners is common. We found two sources that address banner use outside the political domain. Zhou [2] followed banner use in two social movements. He analyzed 143 banners used in 15 environmental protection protests and seven land acquisition disputes. Given his social politics perspective, he noticed that banners contain contentious discourse. He argued that banners can win their audience by transforming individual contention into a collective mission of citizens. The effect of individual initiatives is an interesting topic and we need to see therefore to what extent individual efforts can create a collective response in the digital world of social media, the world which is the source of our data. Klosky and Gitelson [3] and Kohli et al. [4] focused on the functions banners have in tourism and commerce. They used survey data to identify factors that affect the function of banners in marketing, in social and cultural contexts, in relation to an audience and in connection to banner display styles. They concluded that there is no one overwhelming factor that can be identified, but that a variety of factors work together to create the best result. This study, when applied to COVID-19 banners, suggests that a wide variety of factors need to be considered. We therefore need to see what factors are suggested when social media are involved in information processing as is the case in our study.

In the political domain, another important distinction is that between national, regional, and rural authorities, each of these design banners with the intent to influence the population under their authority. As a consequence, the textual contents of banners in the context of the COVID-19 campaign vary in agreement with the background of the local population as we expect to see. There is a shared Marxist ideology across the various Chinese locations, but there can be marked regional differences in the way this ideology is expressed. We found two authors, who, from a political perspective, studied banner use. One of these studied banner production and use, and concluded that banners are a discursive way of energizing and mobilizing the public [5,6]. ‘Mobilizing’ in a lockdown setting, of course, cannot work in a literal sense, it can only take the form of ‘coming together’ and as a result acceptance of governmental directives. The idea of ‘energizing’ is still applicable in a COVID-19 setting and certain banners can indeed ‘energize’ large groups of the population as we will see later. The second author, Xiang [7] collected nearly 3000 banners related to China’s family planning campaigns. His study covered a period of 30 years going back to the 1980s. He made use of Laswell, [8], who developed a model of mass communication, and he concluded that the discourse strategy used in the family planning campaigns rhetorically transformed from propaganda to persuasion. This is a crucial finding and an effect for which we need to scrutinize our data as well.

Finally, we will consider the psychological dimension of banners as well as their discourse status. Sherif [9] and Bellak [10] reported on the social effectiveness of banners from a psychological perspective. They found that certain factors, such as the prestige of the originator of the banners, may influence its effectiveness, and that the change of sentiments and satisfaction with a particular banner may, among other conditions, limit its lifetime. The latter concept in particular is interesting since banners surely have a lifetime, and we need to confirm what the lifetime implications are for our banner study. From a news as discourse perspective [11], banners are one macro-structure, combining both a headline and the lead paragraph. That does mean that world knowledge in banners is concentrated in a limited number of Chinese characters and that readers need to have constructed both a clear mental ‘context model’ and an ‘event model’ of the banner message. Readers of banner messages immediately will understand the goal of the discourse (the context model) as it is so clearly related to the COVID-19 event surrounding them. What we need to focus on therefore are the stylistic choices that banner designers make in order to make their audience understand the implications of their messages. The methods we designed in order to be able to address these stylistic issues, will be addressed now in the next section.

## 2. Materials and Methods

Our survey started at the beginning of the national lockdown, the middle of January 2020, which also was the beginning of the lockdown stage during which a new phase in government-population communication developed. This means that data collection was severely limited and we could mainly focus on the Internet as the source of our data. The data collection did take place between 17 January and 23 February 2020, which covers the early period in which COVID-19 related banners were spreading; in all, a total of 367 examples of banners were collected. These “early COVID-19 banners” were collected from popular Chinese websites by using COVID-19 related keywords. A list of the websites and search engines used is given in Table 1. The main keywords and their frequencies are given in Table 2.

*Main keywords.* This is a list of the most frequently used words showed that the majority (*n* = 179) were related to people’s behavior, with words such as ‘mask’, ‘going out’, ‘staying home’, ‘visiting others’ and ‘quarantine’, in that order of frequency, which immediately makes clear that the government is addressing measures to contain COVID-19. The disease itself was also referred to (*n* = 85) with terms such as ‘epidemic’, ‘virus’ and ‘pneumonia’, also in that order of frequency (Table 2).

*Stylistic and semantic differences in banner language*. In order to find distinctions among the 367 collected “early COVID-19 banners”, we looked at banners as discourse [11,12] and analyzed lexical choices and their frequencies. Syntactic, semantic, and pragmatic analyses, when done, were supported by textual frequency data. In some cases, we draw attention to interactional features such as the use of dialects and internet catchphrases. We also looked at positive or negative implications of our findings, which means that we took note of contextual features that lead to such interpretations and did not take words just at face value. This procedure resulted in a distinction between four banner styles: Official, Gentle, Humorous, and Confrontational. The total number of banners for each of these categories shows that the frequency is in reverse order from ‘confrontational’ with one-in-three representations in the sample to ‘official’ with one-in-six (Table 3). We will discuss the banner language further in the next section.

After these various banners started to circulate on social media, a flurry of comments was produced. A selection of these comments, 84 in total, was collected on the same websites and platforms between 25 January and 10 February 2020. The comments were ordered by the number of likes, reads, reposts, and by their meaning. After the analysis of the various banner styles we will discuss these comments in detail in Section 3 and beyond, by relating the micro level text of samples of comments with the macro level social structures of groups and institutions, that produce them [12] to figure out the social meaning of the public debate over the banners.

## 3. Results

### 3.1. The Language of Early COVID-19 Banners

Banners are, in the first instance, created by authorities, who try in this way to influence the behavior of residents. As indicated in the Methods section, we used the criteria of style, presence of dialect words, narrative perspective and word frequency to identify different banner styles. Our analysis allows a distinction of four types of banners, ‘official’, ‘gentle’, ‘humorous’ and ‘confrontational’, which we will introduce below in that order.

#### 3.1.1. Official

‘Official banners’ are identified as the standard form through which the Chinese government addresses the population. These banners take the form of an ‘instruction’, a ‘directive’ as to how to behave or what to do. An example will help to clarify this. Example 1 below shows the rhetorical pattern of a series of “four-character expressions”, a common formal style, with a large historical background, that relies on shared common ground. In this instance it refers to “confidence (in the government)”, “solidarity (among the people)”, (reliance on) “science” and “policy”.

(1) Jiāndìng xìnxīn, tóngzhōu gòngjì, kēxué fángzhì, jīngzhǔn shīcè

[strengthen confidence; same-boat mutual-aid; science control; precise policy]

“Confidence, solidarity, scientific control, precision strategy” (People.cn, 5 February 2020)

As the example shows, each phrase consists of four characters, which group together as two words as in *Jiāndìng xìnxīn*, ‘[strengthen-confidence], that is, we need to strengthen our confidence (in the government and in a cure). The phrase *tóngzhōu gòngjì*, ‘[same-boat mutual-aid] means we need to work together since we are in the same boat’. *Kēxué fángzhì*, ‘[science control] indicates that science will help us to prevent the disease and find a cure’, and *jīngzhǔn shīcè*, ‘[precise policy] means that our policy is accurate and precise’. As can be understood from this example, this is not an everyday way of speaking and is immediately recognized as an expression based on writing in Chinese characters. Instructions can of course be expressed in normal everyday language as is illustrated by Example 2 which addresses the need for help by medical personnel in the virus-stricken province of Hubei.

(2) Jiānshǒu chūxīn dān shǐmìng, yǒng fù Húběi kàng yìqíng

[hold-fast first-heart take mission, brave go Hubei fight epidemic]

“Stick to your original aspiration and undertake the mission. Bravely go to Hubei to fight

the virus.” (Weibo, 9 February 2020)

The first part of this instruction *Jiānshǒu chūxīn dān shǐmìng*, [hold-fast first-heart take mission] is also an encouragement, which can be translated as given in the example “Stick to your original aspiration and undertake the mission”. In this particular context it refers to medical personnel of other provinces who are encouraged by appealing to their character as ‘brave’ and ‘fighters’-*yǒng fù Húběi kàng yìqíng* ‘[brave go Hubei fight epidemic]-to come to Hubei (Wuhan) to help with the fight against this virus’. This banner is an appeal to steadfastness and courage as in a battle, a way of presenting the situation as in need of help by these members of the population.

The ‘official’ banner type, illustrated by these two examples, represented the smallest number of cases (*n* = 63) in our sample, accounting for not more than seventeen percent or one-in-six of the collected banners (Table 2). Words with a frequency of *n* = 5 or higher in these 63 ‘official’ banners occurred 100 times in total. Excluding the high frequency words reported in Table 2 such as ‘epidemic’ *yìqíng*, ‘quarantine’ *gélí*, ‘mask’ *kǒuzhào*, and ‘virus’ *bìngdú* with 34 occurrences, the remaining group of 66 words are given in Table 4.

Remarkable in this list is the relatively frequent use of *fángkòng* ‘prevention and control’ as in *qiánghuà liánfáng liánkòng* ‘strengthen joint prevention and control’. The term reflects the need of the government to stress its policy measures directed at ‘prevention’ and its goal of controlling the disease in order to ‘win the battle’ *dǎyíng*. ‘Prevention’ is, apart from ‘strong; strengthen’ *qiáng*, *qiánghuà* illustrated in the example just given, also connected to ‘early’ *zǎo*, ‘less’ *shǎo* and ‘diligent’ *qín*. We will give examples of each of these modifiers below, since they nicely make clear the essence of government intent as expressed in these banners.

For ‘early’ *zǎo* we found expressions such as ‘early detect that you are ill’ *zǎo fāxiàn*, ‘early report to the authorities’ *zǎo bàogào*, ‘quarantine yourself early’ *zǎo gélí*, ‘get treatment early’ zǎo *zhìliáo* and ‘see a doctor early’ *zǎo jiùyī*. ‘Less; limit; reduce’ *shǎo* occurred in expressions such as ‘go out less’ *shǎo chūmén*, ‘limit coming together with other people’ *shǎo jùjí*, it is also used in resultative verbs as in ‘reduce contact and infection’ *jiǎnshǎo jiēchù chuánrǎn shǎo*. ‘Diligent’ *qín* was used in ‘preventive’ expressions such as ‘diligently wash your hands’ *qín xǐshǒu*, ‘diligently disinfect’ *qín xiāodú* and ‘diligently ventilate’ *qín tōngfēng*. ‘Strong; strengthen’ *qiáng*, which we already referred to above, we also encountered in expressions such as ‘strengthen protection’ *qiáng fánghù* and ‘enhance physical fitness’ *zēngqiáng tǐzhì*.

In regards to negative terms, *chuányáo* ‘spreading rumors’ is used in opposition to ‘science’ *kēxué* when a banner says ‘rely on science’ *xìn kēxué* and ‘don’t spread rumors’ *bù chuányáo*. As can be expected and as these examples show, official banners are guiding the population to prevent the spread of the COVID-19 virus. Rhetorically, the expressions are serious and authoritative, with carefully chosen words and a commanding tone. They represent, as reported by Han Chengpeng [5], a long-standing government banner style with standardized, careful and formal language.

#### 3.1.2. Gentle

The ‘gentle’ banners, 75 out of the sample of 367, prompt actions in line with the early COVID-19 policies, intended to persuade the public to follow. In that sense, they are not different from the ‘official’ banners. The difference is that the warning tone is ‘gentle’ by appealing to family values and patriotic sensations as a strategy to catch the attention of the banner readers. As in the previous section, we will first give two examples:

(3) Wàichū huílái yào dēngjì, fùlǎo xiāngqīn gǎnxiè nǐ

[out-go back-come must register, elder folks thank you]

“Please announce your presence when you go back to your hometown from other places. Older folks there will be grateful to you for that.” (Weibo, 10 February 2020)

In this example the consequences of the ‘instruction’, ‘register’ if coming back from other places, is followed by gratefulness of a socially respected group ‘older folks’, this way making the message more personal and more humane. The personal sphere is also invoked in Example 4. Where a relation is made between the personal situation and personal behavior, expressed in negative terms as ‘not visiting family members and friends’ and the situation of the country as a whole. If you don’t do the first, you will help the nation:

(4) Bù zǒu qīn, bù fǎng yǒu, bù gěi guójiā tiān máfan

[not go relatives, not visit friends, not give country add trouble]

“Don’t visit your relatives and your friends. Don’t make trouble for the country.” (Toutiao, 1 February 2020)

Word frequency data show that, in these ‘gentle’ banners, there were 126 instances of words with a frequency higher than 5. Of these, four words were part of the top frequency words reported in Table 2, ‘mask’ *kǒuzhào*, *‘*at home*’ zàijiā*, ‘epidemic’ *yìqíng*, and ‘virus’ *bìngdú* in that order with a total of 37 occurrences, which means that, for the ‘gentle’ banners, we can study the nature of 13 frequency words with a total of 89 occurrences. They are listed in Table 5. The first impression, we get from these words is that they are very ‘homely’ *jiālǐ*, expressing ‘coming back’ *huílái* and ‘going out’ *chūmén*, ‘getting together’ *jùhuì* ‘and expressing a relationship with ‘health’ *jiànkāng*. In the context of the COVID-19 epidemic, the movement words, as can be understood, are all presented as negatives, actions to be avoided.

As we look at frequently used modifiers, which we see as indicators of the ‘core values’ the banner makers have in mind, we find three, respectively ‘less or limit’ *shǎo*, ‘good’ *hǎo* and ‘early’ *zǎo*. It is remarkable that the first two, *shǎo* and *hǎo*, had a relatively high frequency of 10 and 9, whereas the third, *zǎo*, was used much less (Table 5). Also, two of these modifiers, *shǎo* and *zǎo*, also occurred in the previous section, which will allow for comparison later. All three will be extensively discussed below.

The ‘less’ *shǎo* examples remind us, first of all, that in 2020 the Chinese New Year started on January 27, that is, in the middle of the COVID-19 crisis. In the ‘gentle’ banners, family relationships are central, and it is no surprise therefore that references to the Chinese New Year did occur, since it was widely perceived as a family distorting happening. One of the examples we found stated, ‘Going out less, staying home more, happy New Year greetings online’, *shǎo chūmén, duō jūjiā, wǎngluò bàinián yuè dàjiā*. Since in Chinese, this is a statement that rhymes, one could think of a translation such as ‘Going out less, staying home more, online greetings are the core’; this way of course would be missing out on the notion of *bàinián*‘celebrating the New Year’. Other examples of the use of ‘less’ are, ‘Dinner together less, relatives will not be gone’, *shǎo chī yī dùn fàn, qīnqī bù huì sàn*. Again, in Chinese this phrase rhymes, so an alternative translation could be ‘Eat together less, family won’t be a mess’. The last example of the three says, ‘Dinner together less, the family relationship will not get weak’, *shǎo jù yī dùn cān, qīnqíng bù huì dàn*, with roughly the same meaning. This banner too contains a rhyme with *cān* ‘meal’ in the opening line and *dàn* ’thin; weak’ in the closing line. Also note the use of *bù huì* ‘will not’ in the latter two examples, which combine a negative advice ‘eat less together’ with the unlikelihood of a potential other negative event, ‘falling apart of family relationships’ expressed as *sàn* ‘scatter’ and *dàn* ‘weak’.

As we found for the ‘less’ examples, the ‘early’ *zǎo* examples bring forward one type of ‘positive’ behavior in order to avoid another form of ‘negative’ behavior or to receive acclaim by doing so. The first example relates ‘self-protection’ and ‘eating behavior’, the often quoted ‘wild animals’ problem, as in: ‘Everyone should take actions on anti-virus early, and wild animals should not be your meal’, *rénrén zuòdào zǎo fángfàn, yěshēng dòngwù mò zuǐchán*. Note the rhyme between *fàn* ‘model’; and *chán* ‘greedy’. The next two examples relate ‘early reporting’ and ‘risk’ in the first of these two and with ‘social gratification’ in the second. ‘If you report your travel history early, the risk of the epidemic will decrease’, *wàidì huílái bàogào zǎo, yìqíng fēngxiǎn jiù biàn xiǎo*, and also, ‘Report early if you return home from an epidemic area, villagers and folks will thank you’, *yìqū fǎnxiāng zǎo dēngjì, fùlǎo xiāngqīn gǎnxiè nǐ*. Both examples also employ rhymes, *zǎo* and *xiǎo* in the first and *dēngjì* and *nǐ* in the second.

With the ‘good’ *hǎo* examples, we meet banners that instruct how to behave and therefore come very close to the style of the ‘official’ banners. The form *hǎo* can be used as the endpoint of a resultative construction, as in *dàihǎo kǒuzhào* [wear-good mask]. Another example of this usage is ‘Stay at home and avoid visiting, the epidemic situation will gradually get better’ *zàijiā xiūxi bié luànpǎo, yìqíng zhújiàn huì zhuǎnhǎo*, while noting the rhyme between *pǎo* ‘to run’ and *hǎo* in *zhuǎnhǎo* ‘turn for the better’. In the remaining examples *hǎo* either occurs in a line or at the end of a line, meaning that either the first part or the second is considered ‘good’ by the writers. We give an example of each. With *hǎo* in the line, the banner states, ‘Keep the house well ventilated, pay attention to hygiene and avoid visiting,’ *jiātíng bǎochí tōngfēng hǎo, jiǎngjiū wèishēng bié luàn pǎo*. ‘Ventilating the home’, which is presented as *hǎo* ‘good’, is followed by two instructions, a positive one about ‘hygiene’ and a prohibition expressed with *bié* ‘do not’, where the latter intends to prohibit *luànpǎo* ‘running around’. The banner also has a rhyme, *hǎo* ‘good’ at the inline position rhymes with *pǎo* ‘to run’ in the closing line. The second example places *hǎo* at the end of the line. The banner states, ‘If you want to have low risk, it is better to stay at home,’ *yàoxiǎng fēngxiǎn xiǎo, háishì jiālǐ hǎo*. The conditional phrase is introduced by ‘want to’, with a rhyme between *xiǎo* ‘little; low (risk)’ and *hǎo* ‘good; better’. ‘Risk avoidance’ is related to a person’s home environment, with the connective *háishì* ‘still is’ projecting the ‘home’ *jiālǐ* as the best place to avoid being contaminated.

#### 3.1.3. Humorous

We chose the term ‘humorous’ for banners which tried to get the banner message across in a more indirect rhetorical technique with a playful tone, this in contrast to the more direct banners we encountered in the previous two sections. The number of examples in this category also increased somewhat with 104 banners out of the 367 samples, a representation of twenty-eight percent. An example of this rhetorical technique which is an indirect warning against the virus and support for the lockdown is given in Example 5:

(5) Nìngyuàn zhǎng diǎn biāo, yě bù wàimiàn piāo

[rather grow bit fat, also not outside wander]

“I would rather add some fat than wander outside.” (Weibo, 31 January 2020)

This message, as a comment on the translation, uses a particular Chinese ‘rather … than …’ (*nìngyuàn … yě bù …*) construction and also uses a rhyme, with *biāo* ‘fat’ in the literal translation line and *piāo* ‘wander’ in the end line. Staying home and little exercise would make people more obese. In this banner, this effect is recognized but presented as still better than wandering outside and by implication getting the virus. Example 6 is another example of this more indirect nature of messaging:

(6) Huí jiā chī zhǔshú de jī, dàjídàlì lì shèqū

[return home eat cook-ripe DE chicken, good-luck benefit community]

“At home eat well cooked chicken, everybody in your community will be lucky.”(Weibo pictures, 7 February 2020)

In this message the link is made between *zhǔshú* ‘well-cooked’ and ‘raw meat’, which is claimed to be a potential origin of the virus and that by doing so you also protect the members of your community. The latter effect was created via a Chinese four-character expression *dàjídàlì* ‘good luck’, which blessings by doing so that is extended to the whole community.

The word frequencies of this set of banners showed that the first five most frequent words were all part of those listed in Table 2. These are ‘mask’ *kǒuzhào*, ‘virus’ *bìngdú*, ‘going out’ *chūmén*, *‘*at home*’ zàijiā* and ‘epidemic’ *yìqíng*, in that order with a total of 84 occurrences, which represents a majority of 60 percent of words with a frequency higher than 5. This is remarkable since these top frequency words did not outperform other frequently used words in either the ‘official’ or the ‘gentle’ banners and suggests that the ‘humorous’ banner set forms a core part of all banners. When we look at the remaining words with a frequency higher than 5 (*n* = 55), these suggest that in the ‘humorous’ banners the family is the orientation point with words such as *guònián* ‘New Year’, *jiālǐ*, ‘at home’, *quánjiā*, ‘whole family’ and *xǐshǒu* ‘washing hands’.

In the remaining modifying words *hǎo* ‘good’, seen in Table 6. stands out with a frequency double that of the remaining modifiers *qín*, ‘diligent’, *shǎo* ‘less’ and *bùyào*, ‘don’t’. This selection and their frequencies also suggest that the ‘humorous’ banners project messages with a rhetorical technique that is different from the two sets discussed above. This becomes especially clear when we consider *guāi*, ‘be good (obedient)’ which also appears in this list, since this word is typically used by parents when talking to children and telling them to be ‘good’ by listening and being ‘obedient’. As an extension of this usage, on banners we found examples such as, ‘Be good, wear a mask!’ *guāi, dàihǎo kǒuzhào*! This banner gives the impression that the government, acting as parents, tells the readers to be nice and obedient and do as told, ‘wear a mask’. Similarly, there is the admonishment ‘Be good, avoid going out!’ *guāi, biāo chūmén*, where *biāo* is a colloquial expression for *bùyào* ‘do not’, the spoken form *biāo* even obtained, through borrowing, its own character, whereas its use suggests that younger people, who too need to wear masks and stay at home, feel more directly involved through this kind of banners. Our third example of *guāi* is a very colloquial word which says ‘Be nice and at home, don’t make a mess, don’t disturb the epidemic prevention.’ *guāiguāi zàijiā mò xiā chuàn, yìqíng fángkòng bié tiānluàn*. The expression *mò xiā chuàn* [don’t run about] ‘don’t run around’ is very colloquial and creates a strong contrast with the formal statements we encountered in the banners discussed in the previous two sections. In this context, it is understood as stay at home and don’t go out.

The relatively high frequency of *hǎo* ‘good’ did not show special construction types, rather it did further illustrate the rhetorical technique directed at changing traditional behavior patterns, some of which are deeply rooted in Chinese culture as we will see shortly. We encountered forms such as *dàihǎo* [wear-good], *zuì hǎo de* [most good DE], *hǎo línjū* [good neighbor], *hǎohàn* [good man], all normal combinations of *hǎo* ‘good’ as modifier. More interesting therefore is the kind of behavior that is encouraged or discouraged. Apart from ‘wearing a mask’, addressed at younger people who are told to listen to their mother, we found ‘staying at home’ as the best protection, not going for a walk, when you ‘have a fever’, and ‘throwing red envelopes out of the window’ instead of bringing them in person. This latter example shows the potential impact of traditional culture for the spread of the virus and we will illustrate that one in full form, ‘Do not enter the building to greet for New Year this time. Toss out the lucky money from the window.’ *jīnnián bàinián bù jìn lóu, hóngbāo zhuāng hǎo rēng xià lóu*. In Chinese culture, it is customary that older people (parents, grandparents) give red envelopes to younger people as a sign of good behavior and blessing for the New Year. The banner discourages New Year visits and handing out red envelopes, rather the youngsters can receive them in this new encouraged way “throw them out of the window’. Of course, we have no knowledge as to the extent to which this was actually done, we were all ‘locked down’ still.

Uses of *shǎo* ‘less’ did show the by now well-known issues of ‘wearing a mask’ and ‘not going out’, the interesting point in these cases is the use of dialect words or expressions, showing local influences in banner construction. In the banner ‘Wear a mask when you go out, don’t visit around.’ *chūmén dài kǒuzhào, shǎo qùqiè còu rènào*, the interesting point is the original dialect expression *qiècòu rènào* ’join the fun’, going out and feel part of the crowd, an effect expressed by *rènào* ‘hot and noisy’. ‘Going out and join the crowd’ is one of China’s leisure time traditions that does not match well with ‘staying at home’ and is the issue that the banner addresses. The rhyme was between *kǒuzhào* ‘mask’ and *rènào* ‘lively’. In the next ‘staying home’ example the rhyme is created through adding of *hāhā* ‘haha’ in the end line in order to make it rhyme with *jūjiā* ‘staying home’ in the in line. ‘Go out less, stay in more, after 14 days have real fun!’ *shǎo chūmén duō jūjiā, 14 tiān hòu lèhāhā*! To make the translation rhyme one could think of ‘Go out less, stay in more, 14 days make fun galore!’ The point of interest remains the use of such funny or everyday language in this banner. The last example too addresses ‘staying at home’ but the word used for ‘staying at home’ is a dialect expression, ‘Stay home more, go outside less!’ *duō zài wūtóu dūn, shǎo qù wàimiàn guàng*. The combination *zài wūtóu dūn* is understood by all Chinese readers, but also recognized as not standard and that gives this banner a nice local flavor, which would be appreciated by local dialect speakers.

Our last frequently used perspective word *qín* ‘diligent’ is focused on ‘washing your hands’ and ‘ventilate the house’. All three examples we give here are rhymes. The rhyme of the second example is created by adding *bàibàibáibai* which can easily be recognized by English speakers as ‘byebye’, which indeed it is. Byebye has become a common greeting among younger people replacing the Chinese *zàijiàn* ‘goodbye’. Here are the three examples with *qín* ‘diligent’, ‘Wash your hands frequently every day, the virus goes away’ *rìcháng qín xǐshǒu, bìngdú ràozhe zǒu*, ‘If you don’t wear a mask, the virus will love you, change the mask frequently, the virus says goodbye’ *Kǒuzhào nǐ bù dài, bìngdú bǎ nǐ ài; kǒuzhào huàn dé qín, bìngdú shuō bàibàibáibai*, and ‘Ventilate and wash your hands frequently, and the coronavirus will slip away’ *tōngfēng huànqì qín xǐshǒu, guānzhuàng bìngdú jiù liūzǒu*, where *guānzhuàng bìngdú* is the Chinese name for the ‘coronavirus’.

#### 3.1.4. Confrontational

Confrontational banners confront readers with the effect of disobedience, it is therefore the reason for which we call these banners confrontational. The language used in confrontational banners has also influenced our title of the paper: “if the virus doesn’t scare you, the banners will”. We found 125 banners out of the 367 collected that matched this criterium, a proportion of thirty-four percent. The choice of words in these banners tends to be colloquial, aggressive and therefore confrontational. An example is a typical confrontational banner, ‘bad behavior’ like ‘not wearing a mask’ is confronted with its alternative ‘being on a ventilator’:

(7) Kǒuzhào háishì hūxījī, nín lǎo kànzhe èr xuǎn yī

[mask or breath-tube, you look-ZHE two choose one]

“A mask or a breathing tube, it’s your call.” (Weibo pictures, 28 January 2020)

The example presents the readers with a choice, do as we tell you or find yourself in the hospital on a ventilator, which one do you prefer? Readers are confronted with a simple choice, ‘mask’ or ‘ICU’. Example 8 extends these personal choices to the level of ‘filial piety’, one of the core values in Chinese culture:

(8) Dàibìng huíxiāng bùxiào érláng, chuánrǎn diēniáng sàngjìn tiānliáng

[bring illness back home non-filial child, infecting dad-mom loose-full heaven-good]

“Returning home with your disease will not make your parents pleased, infecting mom and dad proves you have no conscience.” (Weibo pictures, 10 February 2020)

For sick persons, the choice is not only one for oneself, but one has to face the social implications of disobedience, which in this case means destroying your parents’ life by going home for the New Year festival no matter what and living in shame forever thereafter. These examples make clear that banner messages do not beat about the bush but are direct and confrontational.

Word frequencies further show that the confrontational banners use a different set of words. Top frequency words listed in Table 2 occupied 30 percent of all occurrences, quite a difference with the frequency words in the ‘humorous’ section where they formed the majority. The remaining frequency words are listed in Table 7. Negative words, that is, words that in the context of the COVID-19 virus epidemic have a negative connotation, are strongly represented in this list. These include words like ‘wild animal’, ‘getting together’ and related words like ‘dining’, ‘to gather’ and ‘crowding’. It also makes clear that the virus is ‘the enemy’, and ‘carrying the disease’ is like living in ‘hell’. Death or moral judgements are often used to discourage the public going out or gathering. Bad behaviors like hiding personal infection history, visiting friends and relatives or going out without wearing a mask would lead to terrible consequences such as death or failure of morality. There are few positive frequency words in confrontational banners, which can also reveal the harsh style of the banners.

### 3.2. Banner Comments

The 84 comments we collected are dated between 25 January and 10 February 2020. The first half of these were collected at the end of January with a concentration on January 29 and 30, when 44 percent of the data were collected. The remaining half of the comments were collected in the beginning of February. The comments contained a total of 11,530 characters. The messages varied in length between 12 and 652 words, with an average of 137 words, meaning that the majority of the messages were not that long. In the following, we will detail the sample further as to Internet source, number of words and where appropriate author and support. As to the latter, it is important to know who the writer is and the extent of support for his or her opinion.

#### 3.2.1. Sources and Words

We will start with an overview of the number of contributions among the various Internet sources. As Table 8 shows, *WeChat* stands out with twenty-four comments and a total of more than five thousand characters. *WeiBo* is second with nineteen contributions and almost fifteen hundred characters. In comparison, *Zhihu*, the question-and-answer site, provided more than one thousand characters for four contributions as did the category other, which grouped six sources and contained almost two thousand characters. In the next section we will discuss the content of the collected messages and relate them to the date of the comments.

The most frequent word in the data, not surprisingly, was the term *biāoyǔ* for ‘banner’ with a total of 183 occurrences. We excluded this general term for the frequency wordlist presented in Table 9, since this obviously was the topic of conversation and does not help in understanding the discourse content, our major point of interest. The words in Table 9 are ordered according to descending frequency. The first impression we get from this list is that in the left-hand column the attention is on the ‘epidemic’, on ‘prevention’ and ‘publicity’ but also on ‘hard core banners’, ‘rural communities’ as well as ‘work’ as in *fángyì gōngzuò* ‘epidemic prevention work’ and ‘workers’ *gōngzuò rényuán*. We will see shortly that the most attention by readers is on these issues.

The right-hand column illustrates topics such as the ‘virus’, ‘pneumonia’, ‘purpose’, ‘achieve’ and ‘win’, suggesting that the government policy towards the epidemic is well-known by the commentators. Banner words like ‘grassroots’, ‘ruthless’ and ‘rough’ suggest that there is a conflict between urban and rural areas. To what extent this is played out we will see in the next section.

#### 3.2.2. The ‘Hard-Core’ Banner Debate

On 26 January a Mr Tián posted a ‘hard-core’ *yìnghé* banner comment on *Weibo*:

“I think this kind of hard-core propaganda slogans are needed. Some places are more traditional. Adults and old people don’t listen to it, and don’t listen to young generation’s suggestions at all. The recent epidemic situation is tense, so you should avoid traveling and stay at home. New year greetings are harmful, and special methods should be used during special times.”

He obtained almost nine hundred reads and thirteen likes. A few days later, discussion on the ‘hard-core’ banners had already increased and on January 29 a *Kuaishou* user contributed, “The (hard-core) banners in the countryside hit the nail on the head!”

This post attracted 1.7 million reads and more than 71 thousand likes. From that moment the ‘hard-core’ banners draw the attention of Chinese state media. The People’s Daily, the largest newspaper group in China, posted a *WeChat* commentary article on that day with the title, *Read these banners and you will understand why we need to rely on our people*, saying further, “These ‘provincial’ banners are as rough as landslides, but very “hardcore”, they achieve the purpose of Early COVID-19.”

Since then, over a hundred state media including CCTV (China Central Television), Headline News, People’s Court News forwarded this article or made similar comments, which made the number of comments soar. An example is a contribution from the Weibo site of the People’s Court News, which said:

- “These “earth flavor” banners
- “Relentless” like a mudslide
- But very “hardcore”
- Achieved the purpose of epidemic prevention propaganda
- Let us raise awareness of prevention and wear masks
- Reduce going out
- Together to win this fight against the epidemic!”

This banner was appreciated, read more than twenty-two thousand times, got forty-five likes and was passed on eighteen times. Some of the words used, ‘earth flavor’, ‘relentless’, ‘mudslide’, ‘hardcore’, ‘epidemic prevention’, and ‘propaganda’ keep on returning in other comments. The Chinese words are respectively *tǔwèir*, *wúqíng*, *níshíliú*, *yìnghé*, *fángyì* and *xuānchuán*, of which *níshíliú* had a frequency of nine and just did not make it to the list presented in Table 9. The other words all had the position of top frequency words in that table.

On January 30, a less supportive comment was posted on the *Weibo* site of the Dongfeng (East Wind) Express, which said, ‘Propaganda slogans are too hard-core’, ‘Look at these slogans and banners, and you will understand why you have to rely on the people’:

“The situation of prevention and control of pneumonia caused by the new coronavirus is severe. In some areas, local staff have put up anti-epidemic propaganda. Slogans and banners call on residents to do a good job in epidemic prevention in the form of grounding. These ‘earth-flavored’ slogans and banners are as “relentless” as mudslides, but very “hardcore”. Let us raise awareness of prevention together, wear masks to reduce going out, and win the fight against the epidemic with one heart!”

This less supportive comment was liked two-thousand times and reposted more than five hundred times, confirming that there was a sense of disagreement among the readers. A straightforward oppositional attitude we found in a posting on Weibo from January 30 said:

“Concise and timely slogans can indeed simplify complex truths and make it easier to spread. But we must also realize that this kind of “hard-core slogan” of “barbaric growth” is neither civilized nor scientific. It often carries a destructive meaning, implicitly a certain threat of violence, and even exudes a stale feudal superstition.”

The author continues with emphasis on culture and science and strongly opposes hard-core banner style. It was read more than three hundred times, but did not receive any likes or reposting, clearly showing limited support. This tendency toward a favorite attitude toward ‘down to earth’ banners is confirmed by the data, 62 banners of the 84 or three-fourths of the comments were supportive, whereas two percent were undecided, leaving a minority of close to one-fourth of the respondents to express negative or strongly negative opinions.

#### 3.2.3. Banner Style and Official Media

As reported, state media started to pay attention to the COVID-19 banners and their style. This involvement and positive feedback affected the banner-making process. Banner makers adjusted the banner language style and started to publish more humorous banners. Various provinces of the state media (such as Jilin Youth League) called for Early COVID-19 banners online, encouraging netizens, who are not regular banner makers, to participate in the designing of banners, which characteristically are “easy to understand”, “vivid” and “impressive”. Thereafter state media such as Shenzhen News collected and released new style banners like Examples 9 and 10 below, which use a relaxed language style rarely seen before:

(9) yī mǐ jùlí, nǐ wǒ dōu ānxīn

[one-meter distance, you me both safe]

“One-meter distance makes you and me safe” (Shenzhen News, 11 February 2020)

(10) nìngyuàn kùzi zuò pò, yě bù chūqù rěhuò

[rather trousers sit-broken, YE not out-go court-disaster]

“Would rather sit at home until your pants wear out, do not go out to make trouble.”

(Shenzhen News, 11 February 2020)

We will discuss the implications of this development in the next section.

## 4. Discussion

**Figure 2:**
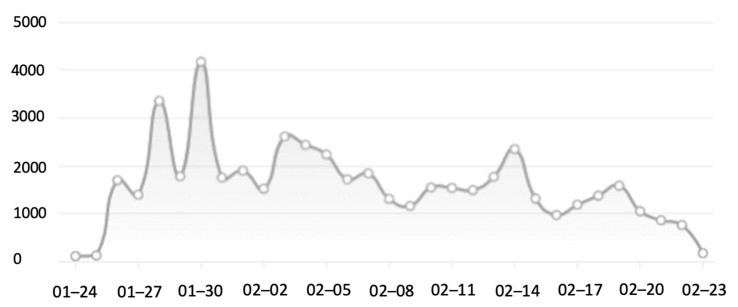
Curve of public attention of early COVID-19 banners. Source: Qingbo database.

The banner data presented so far were collected in the last week of January 2020 and the first week of February 2020, which was during the period of national lockdown. One question therefore is the extent of social media use in this period and particularly the presence of banners and comments on banners on those media. Frequency of social media activities is available from the “Qingbo database” (the Qingbo Big Data database covers various media such as news reports, WeChat, Weibo, forums, and short video platforms. It is a popular big data platform for Chinese news media. It processes data by monitoring and collecting information in real-time from the entire network. Visual analysis technology provides data analysis reports, which can be used to trace and analyze data and information related to emergencies, macro-analyze event attention, and predict its development trend.) and these data show that after the first four banner related articles appeared on January 6, there were very few references until the number of news media articles on January 24 dramatically increased to 121 (Figure 2). Then it reached a peak on January 30 when the number of articles went up to 4165. On February 23 (with an article number of 535) public attention began to decline, went down and remained between 300 to 400 articles until March 31.

WeChat Index data further allow a day-by-day breakdown of the increase in data use in the last days of January. Table 10 shows that from the 27th to the 28th banner related articles increased by forty percent, followed the next day by an increase of more than four hundred percent and after that an increase of another sixty-four percent. After the 30th, data declined by more than fifty percent.

Having positioned banner articles in a wider context, it is time to see to what extent our analysis of the four banner types, *Official, Gentle, Humorous and Confrontational*, relates to word frequencies. The analysis for each banner type was supported by two sets of frequency words, one that represented the banner data as a whole and one for each of the banner types (Table 2 data for each banner type and Table 4, Table 5, Table 6 and Table 7). A comparison between the frequency words of the four banner types shows that indeed each group was supported by a different set of frequency words. This confirmed that each category had different foci, which were respectively ‘anti-COVID-19 policy terms’ such as ‘prevention and control’ and ‘win the battle’, ‘movement’ terms with frequent use of words such as ‘going out’ and ‘getting together’, family terms illustrated by ‘New Year’ and ‘washing your hands’, whereas the confrontational terms focused on hot issues such as ‘wild animal’, ‘getting together’ and ‘dining’, all of which are in the ‘do not’ category but are expressed explicitly in the latter.

A comparison between the two sets of frequency words further showed that the general set occurred most frequently in the Humorous banners and much less in the other banner sets, resulting in a negative correlation between the distribution of the general frequency words and the set specific words of r = −0.16 (*p* < 0.000). We see this as support for our analysis of the banners into different banner categories.

Our second set of data consisted of comments by online readers, who mainly focused on the confrontational banners, the ‘hardcore banners’, as the statistical use data demonstrated. Some of these comments went viral online and triggered a massive public debate. In this process we can distinguish four moments apart from banner recognition: uploading, online spread, online recreation of banners and changes of banner styles in the real world. It is these four phenomena that we will further discuss now at the end of which we will propose a general model that shows the flow of information between these four phenomena.

Physical banners are written on sign boards, fences or walls, or are hung in public areas and seen by readers through on-site instant communication (Figure 1). When such physical banners are photographed and uploaded to the Internet, a copy is created in a new digital environment. Through sharing of information copies spread and if this sharing is intense enough a virtual world is created. We saw above how fast modern technology can help spread a banner and create a new virtual world (Table 10). One could say that digital banners can spread faster than the virus that they are describing. Other than the physical characteristics of the virus, the human mind guarantees that interest in the topic will fade (Figure 2). The banner images multiplied via instant on-site circulation and this caused new events when readers started to comment. Our data showed that the main topics of interest for the readers were on the ‘hardcore’ banners which originated in non-urban areas, where they were stimulated by former CPC (Communist Party of China) practices of propaganda dissemination. A whole set of new words was introduced with clear links to these rural areas with words such as ‘village’, ‘[local taste] earthy’, ‘community’, ‘grassroots’, ‘ruthless’ and ‘rough’. At this point, a national debate developed which placed supporters and their various arguments opposing those who despised the language used on those banners. As we found, a three-quarter majority supported the use of ‘hardcore’ banners to get the COVID-19 message across.

One additional aspect of banner circulation on social media was the effort by some netizens to imitate and recreate similar online banners, usually with an even harsher and funnier content. In Example 11, also a rhyming pattern was created, as was often the case in the official banners. The text read:

(11) Duō hē yī-wǎn biānfú-tāng, huǒzàngchǎng lǐ shuìdé xiāng

[more drink one-cup bat-soup, crematorium in sleep sweet]

“Drink one more bat soup and in the crematorium you sleep.” (Weibo pictures, 26 January 2020)

The word-by-word translation between brackets gives a good impression of this message. The English translation does not repeat the rhyme between *tāng* and *xiāng*, to do that something like “Drink one more cup of bat soup and the crematorium is your coop” would do better. Banner pictures and re-created banners were widely spread through social networks (WeChat, Weibo, etc.), creating a pattern that can be described as a continuous cycle of ‘recognition → upload → forward → comment →forward → upload’ and so on as long as interest in the topic remains.

**Figure 3:**
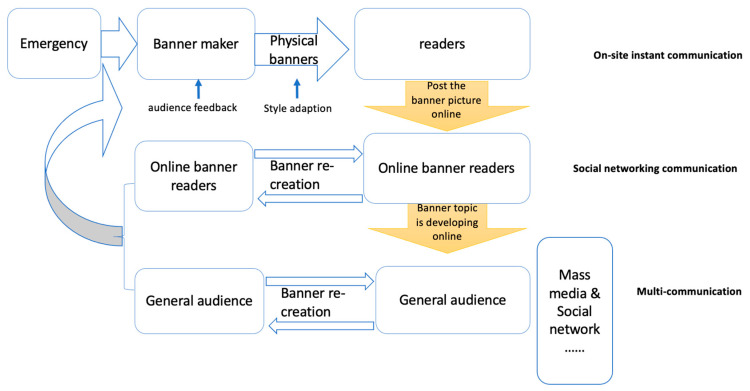
Circulation mode diagram of Early COVID-19 banners.

This process, however, needs a ‘trigger’ to get it to the attention of a wider public and that ‘trigger’ in our data was provided by an official source, the People’s Daily as reported. Since then, over a hundred state media including CCTV (China Central Television), Headline News, People’s Court News and others forwarded this article or made similar contributions, which made the number of comments soar. As a result, Chinese audiences could access banner information through radio, television and newspapers as well as social media such as WeChat and Weibo. Many actively uploaded, forwarded, recreated banners, and made comments. The banner discussion went on through these three channels, real world recognition, social networks and state media. These three communication levels are represented in Figure 3, where it shows that a physical banner reader changes identity when he or she uploads a banner photo and thereby becomes an information source and reaches other readers. The latter can become commentators and re-posters, or themselves take photos of physical banners and post them online creating a feedback loop that way. Official media (written, visual and audio) with a wide audience provide a second channel through which the message spreads and the audience can react. Through this process, some banners were quickly moved onto the public spotlight (see Figure 3).

One final aspect of the feedback mechanism in this model is the change in style of the official media (for instance, CCTV news official Weibo reposted People’s Daily’s popular article *Read these banners and you will understand why we need to rely on our people*, which was reviewed 657 million times, liked 8.6 million times and forwarded 5720 times by 10 February 2020). As a means of official discourse, banners in China are serious in the way they express themselves linguistically. However, as ‘hardcore’ banners received hundreds of millions of reads, likes and reposts, official media started to change their presentation styles too. In a short period of time, Shenzhen News and other media adjusted the style of their COVID-19 banners. ‘Yunnan Daily’, a CPC run newspaper, after the public debate over banner language style, on 22 February released new banners in the more popular style. (Zuò hǎo fánghù gōngchǎng jiàn, gǎn zǒu wēnshén shuǎi mǐxiàn [do well protect factory see, drive away the God of virus, enjoy the rice noodles] Protect yourself well and then see you in the factory. Drive away the God of virus and then enjoy the rice noodles together. (Weibo, 22 February 2020)) In short, the COVID-19 banners influenced official media to switch their language closer to that of the “hardcore” style.

The banner style is the end-result of long-term communication between the banner producer and the audience and, as observed by Guo Linting [13], the social context where banners are generated influences their language style. In addition to their literal meaning, early COVID-19 banners therefore also carry social context information. As observed, hard-core banners were made in rural areas and these banners reflect Chinese rural culture such as the recognition of ‘New Year home coming’, ‘returning to the village’, ‘going around’ and others, while keeping a simple and rough countryside banner style. The banner language in rural China is produced by local grass-root administrators and put on display for farmers [14,15]. For decades, the banners have shown hardcore features which linguistically were simple and rough. Therefore, the language style of early COVID-19 banners fit the rural context, whereas these hardcore characteristics were not noticed until some banners were uploaded to the internet. Through social networking and state media reporting, early COVID-19 banners received a wider audience than that of their rural readers.

The insultingly new style generated huge attention from urban netizens, the not-intended and not-regular readers. The change in the way of dissemination together with loss of the original context triggered great debate. Banner readers interpreted the messages from the perspective of their own social backgrounds. The positive view was that these banners ‘could raise the COVID-19 public awareness’ (Sohu News (https://www.sohu.com/a/371356369_807075)), whereas those who disagreed believed that certain banners are ‘against modern civilization, hurt people’s feelings in this hard time, lower social civilization standards, and damage social harmony’ (Chinese WeChat Official Account of Chinese Language (https://mp.weixin.qq.com/s/cUQs2GCaLO9Gwwiq4rTTLw)).

## 5. Conclusions

The banner as an important official propaganda tool, due to its concise and easy style, has played a crucial role in social organization and coordination for a long time in China. In the context of early COVID-19 communication, we have distinguished four communication styles, ‘official’, ‘gentle’, ‘humorous’ and ‘confrontational’, distinctions which were supported by the statistics in terms of frequency of use. The confrontational form, also called ‘hardcore’ style, attracted most the attention of urban netizens as well as the state media, and inspired the title of our paper: if the virus doesn’t scare you the banner will. The interaction between these two forces resulted in an adjustment of official media communication.

As a particular discourse style, the banner in China plays a role of persuasion, popularization of knowledge, and warning. Early COVID-19 banners were used to warn the public to stay in, since isolation had become an important method of early COVID-19 control and as a result, outdoor activities decreased dramatically. The unique style of early COVID-19 banners attracted wide attention from netizens and for a time formed spectacular banner-oriented enthusiasm. In this process, in addition to its normal purpose, early COVID-19 banners started to function as a source of entertainment. Many netizens considered online activities such as viewing, reposting, and forwarding of comments as a form of indoor entertainment. Online recreated banners and emojis were widely spread, featuring great entertainment, which enhanced netizens’ attention. The decontextualization and extension of COVID-19 banners are linked to the change in circulation mode, from traditional on-site display to decontextualized source of information and that way triggered a new function of entertainment.

Addressing our main questions which were outlined at the beginning of the paper, this paper has shown that in the digital age, the mode of circulation of early COVID-19 banners goes beyond the unidirectional path of on-site instant communication. Facilitated by social networks and mass media, the banner mode upgraded itself twice during the circulation. The upgrades have caused a decontextualization and function extension of the banners during the circulation, and the audience’s feedback on these banners has triggered an adaptive adjustment of the language style of banners.

## Figures and Tables

**Table 1:** List of websites and search engines used for data collection in the 2020 banner survey; 17 January to 23 February 2020.

**Weibo** (Wēibó) www.weibo.com active since 2009 A leading social medium for people to create, share, and discover content online with over 445 million monthly active users by 2018.
**Wechat** (Wēixìn) www.wechat.com active since 2011 The world’s largest free messaging and calling app with over 1 billion monthly active users in 2018.
**Zhihu** (Zhīhū ‘Know all’), https://www.zhihu.com active since 2011 A Chinese question-and-answer website; The number of users had exceeded 220 million and has acculumated more than 30 million questions and 130 million answers by 2018.
**Kuaishou** (Kuàishǒu ‘Quick hand’), https://www.kuaishou.com active since 2011 A Chinese video-sharing mobile app. By 2019, it had surpassed 200 million active daily users.
**TikTok** (Dǒuyīn), www.tiktok.com active since 2016 The destination for short-form mobile videos. As of 2020, it reported around 500 million monthly active users.
**Sohu** (Sōuhú), https://www.sohu.com active since 1996 China’s premier online brand, providing a vast user community with a broad array of choices regarding information, entertainment and communication. It reached 14 million active daily users in 2013.
**Baidu** (Bǎidù), http://www.baidu.com active since 2000 This is China’s Google, one of the largest AI and Internet companies in the world, the third largest website in the Alexa Internet rankings. The number of Baidu’s mobile users reached one billion in 2019.
**Douban** (Dòubàn), https://www.douban.com active since 2005 One of the most influential websites in China that allows users to record information and create content related to films, books, music, recent events, and activities. By 2018, the number of users was around 300 million.
**Toutiao** (Jīnrì Tóutiáo), www.toutiao.com active since 2012 One of China’s largest mobile platforms of Video, Q&A, Fake news moderation, with 550 million daily active users by 2019.
**Netease News**, https://news.163.com active since 1998 One of the most popular news platforms, the number of its mobile users reached 360 million in 2015.

**Table 2:** Frequency of COVID-19 related words; 2020 banner survey; 17 January to 23 February.

Covid-19 Themed	N	Anti-Virus Policy	N
Mask (*kǒuzhào*)	61	Epidemic (*yìqíng*)	44
Going out (*chūmén*)	40	Virus (*bìngdú*)	29
Staying home (*zàijiā*)	35	Pneumonia (*fèiyán*)	12
Visiting (*chuànmén*)	24		
Quarantine (*gélí*)	19		
Total	179		85

**Table 3:** Frequency of banner types in the early 2020 COVID-19 sample.

N/%	Official	Gentle	Humorous	Confrontational	Total
N	63	75	104	125	367
%	17.3	20.4	28.3	34.1	100

**Table 4:** Words with frequencies of 5 and over that appeared in ‘Official’ banners; N = 66.

Positive Words	N	Negative Words	N
*fángkòng*, ‘prevention and control’	14	*shǎo* ‘less’	9
*qín* ‘diligent’	7	*chuányáo* ‘spread rumors’	6
kēxué ‘science’	5		
*jiānjué* ‘be determined’	5		
*dǎyíng* ‘winning the battle’	5		
*jiùyī* ‘seeing a doctor’	5		
*zǎo* ‘early’	5		
*qiáng* ‘strong’	5		
Total	51		15

**Table 5:** Words with frequencies of 5 and over that appeared in ‘Gentle’ banners; N = 89.

Positive Words	N	Negative Words	N
*hǎo* ‘good’	9	*chūmén* ‘go out’	12
*luànpǎo* ‘run around’	5	*shǎo* ‘less’	10
*jùhuì* ‘get together’	8	*Bù huì* ‘will not’	5
*jiànkāng* ‘health’			7
*huílái* ‘come back’			6
*jiālǐ* ‘at home’			6
*rènào* ‘lively’			6
*qīnqíng* ‘affection’			5
*zǎo* ‘early’			5
*gòngxiàn* ‘contribution’			5
Total	62		27

**Table 6:** Words with frequencies of 5 and over that appeared in ‘Humorous’ banners; N = 55.

Positive Words	N	Negative Words	N
*hǎo* ‘good’	11	*shǎo* ‘less’	5
*guònián* ‘New Year’	7	*bùyào* ‘don’t’	5
*xǐshǒu* ‘washing hands’	6		
*jiālǐ* ‘at home’	6		
*guāi* ‘be good (obedient)’	5		
*qín* ‘diligent’	5		
*quánjiā* ‘whole family’	5		
Total	45		10

**Table 7:** Words with frequencies of 5 and over that appeared in ‘Confrontational banners; N = 135.

Positive words	N	Negative words	N
*hǎo* ‘be good’	7	*yěwèi* ‘wild animal’	14
*zìyóu* ‘freedom’	5	*dírén* ‘enemy’	7
		*jùhuì* ‘get together’	7
		*jùcān* ‘dining’	5
		*dìfǔ* ‘hell’	5
		*shàngmén* ‘visit’	5
		*quánjiā* ‘whole family’	5
		*hài rén* ‘harm people’	5
		*dài bìng* ‘sick’	5
		*jùzhòng* ‘crowding’	5
		*huílái* ‘come back’	5
		*jùjí* ‘to gather’	5
		*qīnqī* ‘relatives’	5
Total	12		78

**Table 8:** Number of items for each Internet source used; 2020 Banner survey; N = 11,530.

Internet Source	N	Number of Words N
WeChat	24	5728
Weibo	19	1406
Kuaishou	8	429
Toutiao	6	518
TikTok	5	126
Zhihu	4	1020
Douban	3	56
Sohu	3	320
NetEase	2	111
Other	6	1816
Total	80	11,530

**Table 9:** Words with frequencies of 10 and over that were used in the banner comments; N = 679.

Positive Words	N	Negative Words	N
*fángyì* ‘epidemic prevention’	64	*shífēn* ‘completely’	19
*xuānchuán* ‘publicity’	58	*yìshí* ‘awareness’	18
*yìqíng* ‘epidemic’	52	*jù* ‘get together’	17
*yìnghé* ‘hard core’	45	*jīcéng* ‘grassroots’	16
*héngfú* ‘banners’	39	*bìngdú* ‘virus’	15
*wénmíng* ‘civilization’	33	*fāngshì* ‘patterns’	14
*nóngcūn* ‘rural area’	26	*rénmín qúnzhòng* ‘people’	14
*jiēdìqì* ‘grounded’	22	*wúqíng* ‘ruthless’	14
*tǔwèir* ‘earthy’	22	*cūbào* ‘rough’	13
*shèhuì* community’	21	*zhìhuì* ‘knowledge’	13
*kǒuhào* ‘slogan’	20	*dádào* ‘achieve’	13
*gōngzuò* ‘work’	20	*dǎyíng* ‘to win’	12
		*xiàoguǒ* ‘result’	12
		*wénhuà* ‘culture’	12
		*dàjiā* ‘everyone’	11
		*fèiyán* ‘pneumonia’	11
		*mùdì* ‘purpose’	11
		*chuánbō* ‘propagate’	11
		*zǔjíjīzhàn* ‘blocking action’	11
Total	422		257

**Table 10:** WeChat Index of day-on-day comparison of postings; 2020 banner survey.

Date	Jan 27	Jan 28	Jan 29	Jan 30	Jan 31
**index**	320,246	444,980	2,376,330	3,894,946	1,868,300
**day-by-day in/decrease (%)**	1.45	38.95	434.03	63.91	52.03
